# *DUOX2*/*DUOXA2* Mutations Frequently Cause Congenital Hypothyroidism that Evades Detection on Newborn Screening in the United Kingdom

**DOI:** 10.1089/thy.2018.0587

**Published:** 2019-06-03

**Authors:** Catherine Peters, Adeline K. Nicholas, Erik Schoenmakers, Greta Lyons, Shirley Langham, Eva G. Serra, Neil J. Sebire, Marina Muzza, Laura Fugazzola, Nadia Schoenmakers

**Affiliations:** ^1^ Department of Endocrinology, Great Ormond Street Hospital for Children, London, United Kingdom.; ^2^ University of Cambridge Metabolic Research Laboratories, Wellcome Trust-Medical Research Council Institute of Metabolic Science, Addenbrooke's Hospital, Cambridge, United Kingdom.; ^3^ Department of Human Genetics, The Wellcome Trust Sanger Institute, Hinxton, United Kingdom.; ^4^ Department of Laboratory Medicine, Great Ormond Street Hospital for Children, London, United Kingdom.; ^5^ Division of Endocrinology and Metabolic Diseases, IRCCS Istituto Auxologico Italiano, Milan, Italy.; ^6^ Department of Pathophysiology and Transplantation, University of Milan, Milan, Italy.

**Keywords:** congenital hypothyroidism, *DUOX2*, *DUOXA2*, dyshormonogenesis, gland *in situ*

## Abstract

***Background:*** The etiology, course, and most appropriate management of borderline congenital hypothyroidism (CH) are poorly defined, such that the optimal threshold for diagnosis with bloodspot screening thyrotropin (bsTSH) measurement remains controversial. Dual oxidase 2 (*DUOX2*) mutations may initially cause borderline elevation of bsTSH, which later evolves into significant hypothyroidism on venous blood measurement. It was hypothesized that mutations in both *DUOX2* and its accessory protein *DUOXA2* may occur frequently, even in patients with borderline bsTSH elevation, such that higher diagnostic thresholds in bsTSH screening may fail to detect such cases, with consequent risk of undiagnosed neonatal hypothyroidism of sufficient magnitude to require thyroxine therapy. This study aimed to investigate the frequency and characteristics of *DUOX2* and *DUOXA2* mutations in a borderline CH cohort.

***Methods:*** A cross-sectional study of patients with borderline CH was undertaken at Great Ormond Street Hospital, a tertiary British pediatric center. *DUOX2* was sequenced in 52 patients with a bsTSH of 6–19.9 mIU/L, venous TSH of >25 mIU/L, and eutopic thyroid gland *in situ*. *DUOXA2* was sequenced in *DUOX2* mutation-negative cases, and novel *DUOXA2* mutations were functionally characterized.

***Results:*** A total of 26 (50%) patients harbored likely pathogenic mutations in *DUOX2* (*n* = 20; 38%) or *DUOXA2* (*n* = 6; 12%), including novel gene variants (*DUOX2*, *n* = 3; *DUOXA2*, *n* = 7). Two recurrent *DUOX2* mutations (p.Q570L, p.F966Sfs*29) occurred frequently in population databases (MAF ≥0.01). Despite bsTSH being <10 mIU/L in 46% of *DUOX2* and *DUOXA2* mutation-positive cases, venous free thyroxine levels in these patients were in the moderate CH range (*M* = 9.3 pmol/L, range <3.9–15.8 pmol/L),

***Conclusions:*** Targeted *DUOX2* and *DUOXA2* sequencing in a borderline CH cohort has a high diagnostic yield. These findings might argue for a lowering of bsTSH thresholds, but follow-up studies are required to assess whether cases with borderline bsTSH harboring *DUOX2*/*DUOXA2* mutations will benefit from an early diagnosis and subsequent levothyroxine treatment.

## Introduction

Primary congenital hypothyroidism (CH) is the commonest neonatal endocrine disorder, for which prompt diagnosis and initiation of levothyroxine (LT4) therapy is required to prevent irreversible neurodevelopmental delay. The British newborn screening program for CH was introduced in 1983 and has been a major public-health success, with near elimination of profound physical and cognitive impairments associated with delayed treatment (1,2). Recent improvements in sensitivity of thyrotropin (TSH) assays and reports of missed cases have prompted lowering in bloodspot TSH (bsTSH) thresholds for investigation. However, despite a recommended threshold of 10 mIU/L by the national screening program in the United Kingdom, cutoff thresholds used in bsTSH screening continue to be highly variable and center-specific, ranging from 5 to 10 mIU/L. Recent British data suggest that a bsTSH threshold of 8 mIU/L may be optimal (3). However, arguments against universal lowering of bsTSH screening thresholds include the suggestion that some babies with mildly elevated TSH do not have “true” CH (4), as well as controversy regarding the benefits of treating mild or subclinical CH.

Although prevention of neurodevelopmental impairment is the main reason for identification of CH, guiding sensitivity and specificity of the screening program, associative data between borderline bsTSH elevation and IQ have previously been lacking. Two recent studies addressing this question have yielded discordant results. A study in Belgian preschool children found no association between TSH concentration (0.1–15 IU/L) and developmental outcomes when adjusting for confounding factors (5). However, a large Australian epidemiologic record linkage study did find a relationship between higher TSH levels and poorer intellectual attainment in children with a TSH above the 75th centile of the population, which likely equates to a bsTSH concentration below the lower limit in current CH screening programs in the United Kingdom (6). Thus, this study raises concerns about neurodevelopmental implications in borderline CH cases that may be missed using current bsTSH screening criteria.

Primary CH results from either abnormal thyroid development (thyroid dysgenesis [TD]) or inadequate thyroid hormone biosynthesis from a eutopic thyroid gland *in situ* (GIS CH). Although early studies reported that 80% of CH was due to TD (1,2), lower bsTSH thresholds have resulted in a doubling of the incidence of CH, largely due to GIS CH (7). In mild or subclinical GIS CH, genetic studies have usually analyzed *TSHR*, identifying causal mutations in up to 29% of cases (8). However, mutations in *DUOX2*, the NADPH oxidase that generates hydrogen peroxide (H_2_O_2_) essential for organification of iodide, and its accessory protein DUOXA2 are also likely contributors, and the incidence of *DUOX2/DUOXA2* mutations in borderline CH has not been fully evaluated in a large cohort (9–11). Crucially, since *DUOX2* mutations may be associated with borderline bsTSH elevation but markedly subnormal venous free thyroxine (fT4) levels at confirmatory testing, higher bsTSH screening cutoffs may not detect these cases, resulting in overt hypothyroidism that remains untreated neonatally, although such dysfunction can resolve later in childhood (12).

*DUOX2* mutations are a well-recognized cause of CH in Caucasian patients and a major contributor in the Far East, with mutations identified in up to one third of GIS CH cases, depending on selection criteria (10–12). *DUOXA2* mutations are rare (an NCBI PubMed search revealed 16 CH-associated mutations in addition to one whole gene deletion), although an incidence of 7% was reported in a recent study of unselected Korean CH cases, largely due to a single recurrent mutation (p.Y138*) (9,11,13). This study investigated the frequency of *DUOX2/DUOXA2* mutations in borderline bsTSH GIS CH cases from a single British center, and evaluated the biochemical characteristics and requirement for LT4 treatment in this cohort. Given the relative rarity of *DUOXA2* mutations, functional studies were also undertaken to investigate the effect of novel *DUOXA2* mutations on DUOX2-mediated H_2_O_2_ production.

## Methods

The study was approved by Cambridge South REC (MREC 98/5/24) and includes additional measurements undertaken as part of routine clinical follow-up with consent from patients and/or next of kin.

### Study criteria

Between January 1, 2013, and December 31, 2015, 361,839 babies were screened for CH by the North Thames newborn screening laboratory based at Great Ormond Street Hospital using a lower borderline bsTSH cutoff of 6 mIU/L. Infants referred with an initial bsTSH between 6 and 19.9 mIU/L and a second bsTSH >6 mIU/L one week later were selected. Further inclusion criteria included initial venous TSH (vTSH) >25 mIU/L, LT4 treatment, negative antithyroid peroxidase antibody testing, and a normally positioned thyroid gland assessed by technetium (Tc-99m) pertechnetate scintigraphy. Exclusion criteria included birth at <32 weeks of gestation, significant comorbidities, or maternal and infant thyroid autoantibodies.

Of the 519 children identified in this time interval, 83 were eligible for inclusion (based on the inclusion and exclusion criteria set out above), and 52 infants were recruited. Eleven infants were followed up elsewhere; one family declined and 19 did not provide parental consent and/or DNA samples in the time frame of the study.

*DUOX2* was screened in all cases, and in cases where no mutation was identified, the study proceeded to *DUOXA2* sequencing. Further details of the study cohort and biochemical assays are provided in the Supplementary Data.

### Genetic sequencing

Genomic DNA was extracted from peripheral blood leukocytes using standard techniques. In the probands, all 34 *DUOX2* and all six *DUOXA2* exons and exon/intron boundaries were amplified by polymerase chain reaction (PCR) using specific primers (available on request). Family members were genotyped for mutations identified in the CH cases by amplifying and sequencing the relevant exon. PCR products were sequenced using the BigDye Terminator v3.1 Cycle Sequencing Kit (Applied Biosystems, Foster City, CA) and 3730 DNA Analyzer (Applied Biosystems). The variants listed in this study are described using the systematic nomenclature approved by the Human Genome Variation Society (HGVS; www.hgvs.org/mutnomen). Nucleotide numbering starts from the A (+1) of the translation initiation codon (ATG) of the NCBI reference sequence NM_014080.4 (*DUOX2*) and NM_207581.3 (*DUOXA2*). Amino acid residues are numbered according to the NCBI reference sequence NP_054799.4 (DUOX2) and NP_997464.2 (DUOXA2).

Mutations in *DUOX2* were classified as pathogenic if there was supporting literature, or if they affected the protein coding sequence and were predicted to be pathogenic *in silico*. *DUOXA2* mutations were assessed *in vitro* for their effect on DUOX2-mediated H_2_O_2_ production (see Supplementary Data for further details).

### Statistics

Clinical characteristics of infants who were *DUOX2/DUOXA2* mutation positive or negative were compared using comparison of proportions/chi-square test or Fisher's exact test. Distributions of continuous variables, including birth weight, bsTSH, vTSH, and fT4, across groups were examined using analysis of variance (ANOVA) for mutation-positive, mutation-negative, and not-tested groups, and Mann–Whitney *U*-test or an unpaired two-tailed Student's *t*-test for paired comparisons. Comparison of H_2_O_2_ production by cells expressing mutant *DUOXA2* compared to those expressing wild-type *DUOXA2* were performed using one-way ANOVA with Tukey's *post hoc* test.

### *DUOX2*–*DUOXA2* functional analysis

#### H_2_O_2_ generation by the DUOX2–DUOXA2 complex in HeLa cells

HeLa cells were transiently transfected with HA-DUOX2, DUOXA2-Myc/His (kind gifts from Dr. S. Refetoff, University of Chicago) (14) and 50 ng BOS β-Gal expressing plasmids. Extracellular H_2_O_2_ accumulation was assayed by addition of Amplex Red reagent (Thermo Fisher Scientific, Waltham, MA) to the medium and measurement of fluorescence using an Infinite M1000 Pro microplate reader (Tecan, Männedorf, Switzerland). Values were adjusted for transfection efficiency quantified by a β-Gal assay. Results were an average of at least 10 experiments, normalized to wild-type DUOX–DUOXA2 set to 100% (see Supplementary Data for further details).

#### DUOX2–DUOXA2 expression analysis by Western blot

HEK293 cells were transiently transfected with NH_2_-terminal Myc-epitope tagged *DUOXA2* expression vectors. Cell lysate was then run on a 10% sodium dodecyl sulfate polyacrylamide gel electrophoresis gel (Thermo Fisher Scientific), and expression of *DUOXA2* was analyzed by Western blotting using anti-Myc antibody (9E10; Santa Cruz Biotechnology, Dallas, TX; see Supplementary Data for further details).

## Results

### Cohort characteristics and genetic findings

A total of 52 cases (27 males) were recruited, for whom clinical characteristics are summarized in Tables 1 and 2 and Supplementary Tables S1–S3; cases who fulfilled recruitment criteria but did not participate shared similar clinical and demographic features. Twenty-six (50%) cases harbored mutations in either *DUOX2* (*n* = 20; 38%) or *DUOXA2* (*n* = 6; 12%), collectively termed mutation-positive cases. Birth weight, gestational age, ethnicity, and venous fT4 levels were similar in mutation-negative and -positive subsets, and the entire cohort was greatly enriched for children of Asian/Asian British ethnicity, comprising 53% cases.

**Table 1.** Characteristics of Cohort Comparing Mutation-Positive, Mutation-Negative, and Untested Cases

	*Mutation positive*		*Mutation negative*	*Not tested*^a^	p*-Value*
	DUOX2 *(*n* = 20)*	DUOXA2 *(*n* = 6)*	*(*n* = 26)*	*(*n* = 31)*	*Mutation positive versus mutation negative*
*Ethnicity*					
1 White	2	1	6	5	0.3
2 Mixed	1	1	2	2	1
3 Asian/Asian British	11	4	13	19	0.6
4 Black/Black British	1	0	3	2	0.3
5 Other	5	0	2	3	0.2
Consanguineous parents	3	0	5	5 (4 not recorded)	0.7
M:F	8:12	4:2	15:11	14:17	0.5
Mean 1st bloodspot, mIU/L TSH (range)	10.9 (6.2–18.8)	12.2 (7.9–14.6)	11.7 (6.3–18.3)	11.3 (6.8–19.5)	0.7
Mean venous TSH, mIU/L (range)	66.1 (29.8–150)	77.8 (29.3–125)	42.5 (25.0–100)	54.7 (25.9–224)	**0.01**
Mean venous fT4, pmol/L (range)	9.5 (<3.9–15.8)	8.5 (5.5–11.8)	11.1 (<3.9–15.6)	10.1 (<3.9–17.3)	0.2
Mean birth weight (g)	2997	3048	3117	2996	0.7
Confirmed transient CH	8 (40%)	2 (33%)	10 (38%)	N/A	1

Bold indicates *p* < 0.05.

^a^ Not tested due to practicalities of obtaining consent or DNA in study period or lost to follow-up (one declined). Confirmed transient CH: cases who had ceased LT4 treatment by the end of the study. Bloodspot TSH reference range <6 mIU/L. Venous fT4 reference range 12.5–24.6 pmol/L. Comparisons of distributions between groups were performed with a Mann–Whitney *U*-test, and comparisons of proportions were performed using the chi-square test/Fisher's exact test.

M, male; F, female; TSH, thyrotropin; fT4, free thyroxine; CH, congenital hypothyroidism; LT4, levothyroxine.

**Table 2.** Summary of Treatment Outcomes

*Parameter*	*Mutation positive*	*Mutation negative*	p*-Value*
Confirmed transient CH or reducing dose of levothyroxine (% cohort)	14 (54%)	10 (38%)	0.4
Outcome unknown or likely permanent CH (% cohort)	12 (46%)	16 (62%)	
Mean dose LT4, μg/kg (range), number of cases evaluated	2.18 (0.8–4.2), *n* = 11	2.15 (1.3–4.7), *n* = 16	0.9

Summary of treatment outcomes for those individuals in mutation-negative and mutation-positive cohorts in whom a trial of weaning off LT4 treatment was attempted, and mean LT4 dose in individuals remaining on LT4 at the end of the study. *p*-Values were computed using Fisher's exact test for treatment outcomes and an unpaired two-tailed Student's *t*-test for comparison of the mean dose of LT4.

**FIG. 1.**
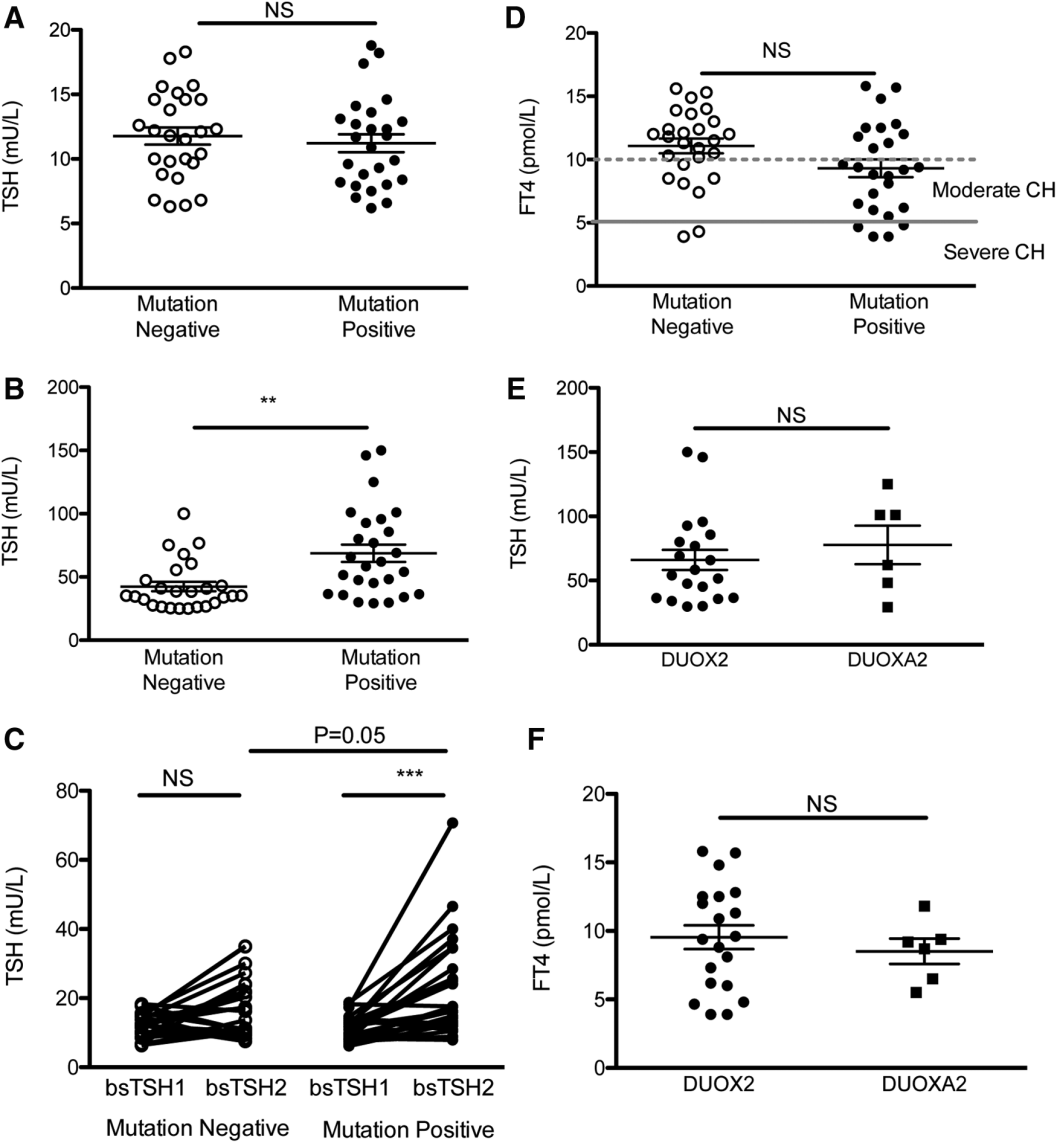
Graphs showing first screening (**A**) bloodspot thyrotropin (bsTSH), and (**B**) venous TSH (vTSH) in cases with (positive) and without (negative) mutations in *DUOX2* or *DUOXA2*. First and second bsTSH readings in mutation-positive and -negative cases are shown in (**C**), and venous free thyroxine (fT4) levels in mutation-positive and -negative cases are shown in (**D**). European Society for Paediatric Endocrinology cut points for moderate (<10 pmol/L) and severe (<5 pmol/L) congenital hypothyroidism (CH) are also delineated; fT4 values <3.9 pmol/L are plotted as 3.9 pmol/L. Comparison of (**E**) vTSH and (**F**) venous fT4 in patients with mutations in *DUOX2* and *DUOXA2*. *p*-Values denote results of a Mann–Whitney *U*-test. Bars denote mean and standard error of the mean. NS, not significant (*p* ≥ 0.05). **p < 0.005; ***p < 0.0005.

As expected from the selection criteria, mean bsTSH values were similar in mutation-positive and -negative cases, but mutation-positive cases had greater TSH elevation on venous confirmatory testing (Fig. 1A and B). bsTSH significantly increased on the second bsTSH measurement in the mutation-positive cohort, and there was a tendency for the absolute value of the second bsTSH to be higher in the mutation-positive cases (mean mutation-positive second bsTSH 23.1 ± 2.91 mIU/L, mutation-negative second bsTSH 16.6 ± 1.7 mIU/L, *p* = 0.05; Fig. 1C). Sixty-two percent of mutation-positive cases and 27% of mutation-negative cases had venous fT4 levels satisfying European Society for Paediatric Endocrinology criteria for moderate-severe CH, with venous fT4 measuring <5 pmol/L in four mutation-positive and two mutation-negative cases (Fig. 1D). Despite significantly subnormal venous fT4 levels, 12 (46%) mutation-positive cases and 12 (46%) mutation-negative cases had at least one bsTSH level <10 mIU/L, and would therefore have evaded detection using the conventional British newborn screening program center lower borderline cutoff value of 10 mIU/L (Fig. 1D and Supplementary Tables S1 and S2). Biochemistry was comparable in cases harboring either *DUOXA2* or *DUOX2* mutations (Fig. 1E and F). None of the patients had clinically evident goiter. However, it was not possible to evaluate definitively whether the thyroid was enlarged, since Tc-99m scanning rather than ultrasonography was performed to establish the presence of a thyroid gland in all except two cases, and radionuclide studies are poor markers of thyroid size.

Despite significantly subnormal venous fT4 levels, the duration of CH in both mutation-positive and -negative cases was most likely transient. Follow-up of the 26 mutation-positive cases revealed that 14 (53.8%) cases had confirmed transient CH or were being treated with a reduced dose of LT4 (Tables 1 and 2 and Supplementary Tables S1 and S2). A similar percentage of mutation-negative cases also had confirmed transient CH (10/26 cases; Table 2; Fisher's exact test *p* = 0.4 showing no significant difference). Eight mutation-positive and nine mutation-negative cases were deemed too young for LT4 withdrawal by the end of the study (Supplementary Tables S1 and S2). The mean LT4 dose was similar in both mutation-positive and -negative evaluable cases (Table 2).

Parental genotype was obtained in all except five mothers and seven fathers (Supplementary Table S3 and Table 3). Maternal thyroid function tests were routinely checked at the time of diagnosis of CH in their offspring postpartum. Only two mothers were mutation negative, which precluded comparison of thyroid function in wild-type and heterozygous individuals. However, thyroid biochemistry was available for 25 mothers, none of whom were on LT4 treatment at the time of their child's referral for CH. One mother had a mildly subnormal fT4 level that spontaneously normalized without treatment (case 6, DUOX2 p.R764W heterozygote) and one mother (case 25, DUOXA2 p.L204P heterozygote) subsequently developed non-autoimmune hypothyroidism, for which she remains on LT4 treatment. Four infants also had siblings who were diagnosed with CH following neonatal screening; two affected siblings were genotyped and were either *DUOX2* mutation compound hetero-or homozygotes (Table 3). Paternal thyroid biochemistry was not available.

**Table 3.** Summary of Parental Genotypes and Maternal Thyroid Biochemistry

*Case (sex)*	*Mutation*	*Paternal genotype*	*Maternal genotype*	*Maternal TSH (<4.0 mIU/L)*	*Maternal fT4 (10.2–20.6 pmol/L)*
DUOX2 *mutation*					
1 (M)^a,b^	p.R411K (Het)	WT	Het	1.4	13.9
	p.R1084Q (Het)	Het	WT		
2 (M)	c.3693 + 1G>T (Het)	WT	Het	1.9	14
3 (F)^c^	p.F966Sfs^*^29 (Het)	NA	Het	1	16.3
4 (F)	p.Q570L (Het)	NA	NA	1.2	14.8
	p.F966Sfs^*^29 (Het)	NA	NA		
5 (F)	p.L129F (Het)	WT	Het	1.5	17
6 (F)^d^	p.R764W (Het)	NA	Het	3	**9.4**
7 (M)	p.F966Sfs^*^29 (Het)	WT	Het	0.6	16.1
8 (F)	p.Q570L (Hom)	Het	Het	2.1	12.8
9 (F)	p.Q202Tfs^*^99 (Hom)	Het	Het	1.1	13
10 (M)^e^	p.E879K (Het)	WT	Het	1.3	12.9
11 (F)	p.Q570L (Het)	WT	Het	0.3	17.8
12 (F)^a^	p.A239T (Het)	WT	Het	NA	NA
	p.Q570L (Het)	Het	WT		
13(M)^a^	p.Q570L (Het)	Het	WT	2.8	12.2
	p.E1469K (Het)	WT	Het		
14 (M)	p.G488R (Het)	WT	Het	0.9	15.4
15 (M)^f^	p.R683L (Het)	Het	WT	0.3	16.5
	p.L1343F (Het)	Het	WT		
16 (M)	p.F966Sfs^*^29 (Hom)	NA	NA	1.7	12.5
17 (M)	p.Q202Rfs^*^93 (Het)	Het	WT	1.7	12.5
18 (F)	p.Q202Tfs^*^99 (Hom)	Het	Het	2.1	16.7
19 (F)^a,e^	p.Q570L (Het)	WT	Het	1.7	12.5
	p.E1314K (Het)	Het	WT		
20 (M)	p.S965L (Het)	NA	NA	3.5	12.6
DUOXA2 *mutation*					
21 (M)	p.E128^*^ (Het)	Het	WT	1.1	13.1
22 (M)	p.N121_E122delinsK (Het)	NA	NA	1.5	13.1
23 (F)^a^	p.G264R (Het)	Het	WT	1.8	16.7
	p.L298Hfs^*^21 (Het)	WT	Het		
24 (M)	p.V78M (Het)	NA	NA	2.8	12.9
25 (F)^a,g^	p.W76C (Het)	Het	WT	**4.0**	13.5
	p.L204P (Het)	WT	Het		
26 (M)	p.E128^*^ (Het)	WT	Het	1.7	12.7

Maternal thyroid function tests were all checked postpartum at the time of diagnosis of CH in their offspring. Bold values denote results outside the reference range.

^a^ Confirmed compound heterozygote.

^b^ Sibling with transient CH, also compound heterozygous for p.R411K and p.R1084Q.

^c^ Sibling with transient CH, homozygous for p.F966Sfs^*^29.

^d^ Subnormal maternal fT4 postpartum, which resolved without treatment.

^e^ Sibling with CH for whom genetic testing was not undertaken.

^f^ Maternal iodine supplementation during pregnancy.

^g^ Maternal levothyroxine treatment subsequently commenced for hypothyroidism (thyroid function tests at diagnosis not available).

Het, heterozygous; WT, wild type; Hom, homozygous; NA, not available.

### DUOX2 mutations

**FIG. 2.**
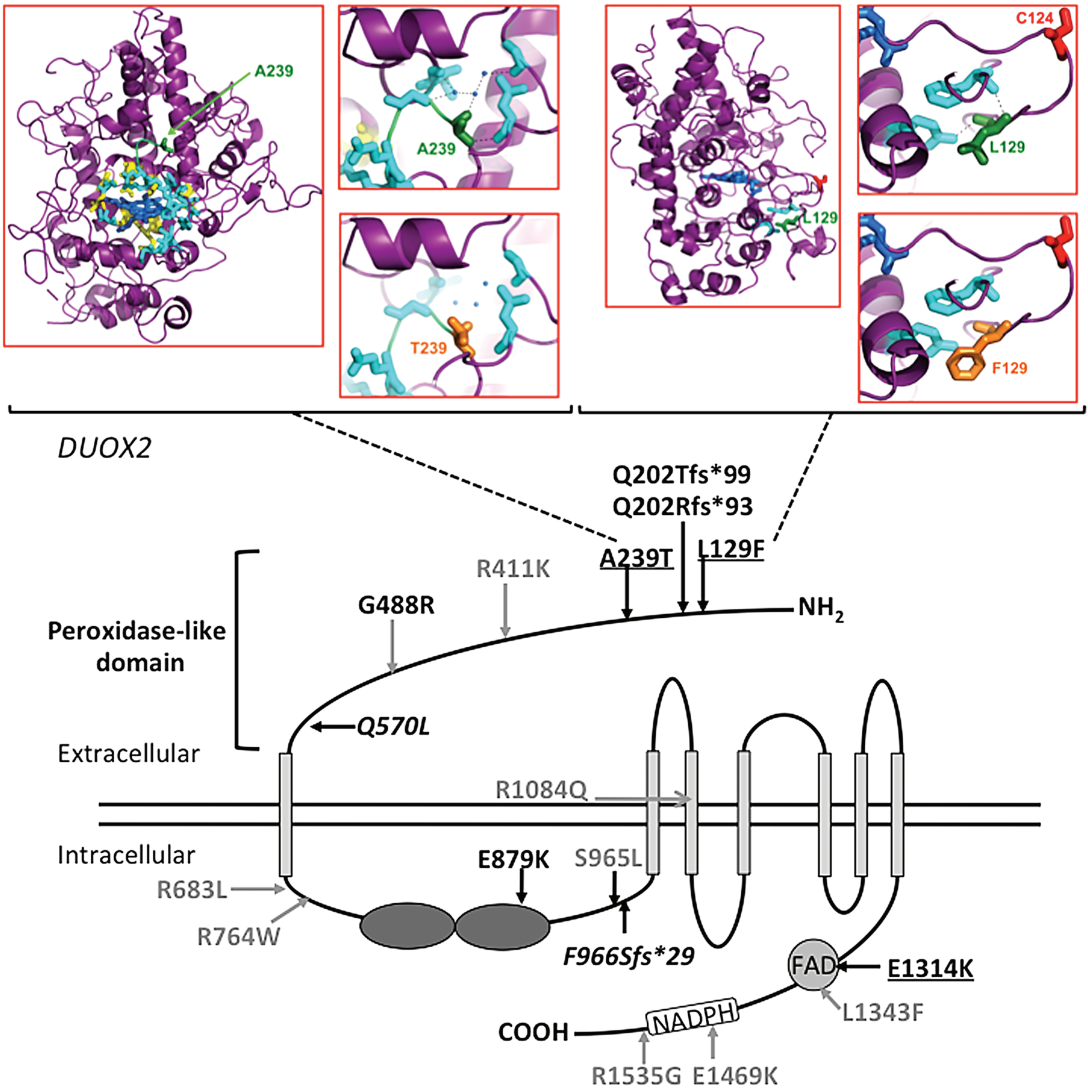
Schematic showing *DUOX2*, functional domains, and the position of mutations identified in this study. Known CH-associated mutations that have not undergone functional characterization are shown in gray. Known CH-associated truncating mutations or missense mutations shown to be pathogenic *in vitro* are shown in black. Novel mutations are shown in black and underlined. Recurrent mutations affecting more than two cases in this study are shown in italics. The model was constructed using data from the InterPro database. Homology modeling has been used to predict the consequences of the novel mutations A239T and L129F. A239 (green) is small and apolar, while T (orange) is large and polar and disrupts local polar contacts (blue) and spacefill, probably disrupting backbone H-bonds between A239 and H_2_0. L129 (green) and F (orange) are both hydrophobic, but F is significantly larger and does not fit in the position of L129 that will affect the local structure. C124 (red) is situated in a loop that may be repositioned with the L129F mutation, and since C124 is reported to form a disulfide bridge with C1162, this may destabilize *DUOX2*. Color images are available online.

Twenty cases harbored confirmed or likely pathogenic *DUOX2* mutations, including 11 with monoallelic mutations, eight with biallelic mutations, and one harboring two different variants for which parental inheritance could not be investigated (Fig. 2 and Supplementary Tables S1 and S3). Severity of thyroid dysfunction did not correlate with the number of mutant *DUOX2* alleles (monoallelic mutations: mean ± standard error of the mean vTSH = 63.5 ± 10.1 mIU/L; biallelic mutations: mean ± standard error of the mean vTSH = 74.1 ± 13.5 mIU/L, *p* = 0.54). Six mutations were known truncating mutations or missense mutations associated with decreased H_2_O_2_ *in vitro* (see Human Gene Mutation Database, HGMD Professional v18.2 for details, reviewed in Muzza and Fugazzola) (15). An additional eight missense mutations had not been functionally characterized, but had a minor allele frequency of <0.01 in the Exome Aggregation Consortium database (ExAC; Cambridge, MA), and had previously been associated with CH. With the exception of the splice site mutation c.3693 + 1G > T for which predictions were not available, all were predicted to be deleterious by SIFT and/or at least possibly damaging by Polyphen-2. Three missense mutations were novel, involving the peroxidase-like domain (c.385C > T, p.L129F, c.715G > A, A239T) and FAD domain (c.3940G > A, p.E1314K). p.L129F occurred in isolation, whereas p.A239T and p.E1314K occurred in compound heterozygosity with the known pathogenic mutation p.Q570L. Although all three mutations were predicted to be disease-causing by MutationTaster, p.E1314K was predicted to be deleterious by SIFT and probably damaging by PolyPhen-2, but p.L129F and p.A239T were classified as tolerated and benign by SIFT and PolyPhen-2, respectively (Supplementary Table S3). Homology modeling was therefore undertaken for the latter two mutations, which also suggested that they were likely to be pathogenic (Fig. 2). Alanine (A) is small and apolar, whereas threonine (T) is large and polar and likely to disrupt polar contacts in DUOX2 A239T, resulting in destabilizing local structure/function. Leucine (L) and phenylalanine (F) are both hydrophobic. However, F is significantly larger than L and unable to occupy the same space and molecular position as L129, resulting in a perturbed local structure in *DUOX2* L129F, possibly with repositioning of C124 and disruption of the C124-C1162 disulfide bridge, which may consequently destabilize the DUOX2 protein (Fig. 2). In view of these findings, L129F and A239T were also classified as mutations for the purposes of this study. Two mutations were recurrent: c.1709A > T, p.Q570L (6 cases, MAF 0.01, South Asians) and c.2895_2898delGTTC, p.F966Sfs*29 (4 cases, MAF 0.01, European Finnish population). Both occurred with MAF ≥0.01 in specific ethnic groups in the ExAC population database.

Three *DUOX2* single nucleotide polymorphisms (SNPs)—p.P138L (rs2001616), p.L1067S (rs269868), and p.H678R (rs57659670)—have variably demonstrated enrichment in CH cases, although conflicting functional characterization and prevalence data have led to ambiguity regarding their significance (12,16,17). Frequencies of these SNPs within the cohort were computed (allele frequencies p.P138L: 0.87; p.L1067S: 0.17; and p.H678R: 0.1). However, within the ExAC database, allele frequencies at these positions may vary significantly, depending on ethnicity: p.P138L 0.4 (African)–0.9 (South Asian), p.H678R 0.05 (East Asian)–0.38 (African), and p.L1067S 0.06 (East Asian)–0.7 (African). The small size and mixed ethnicity of the cohort precluded meaningful comparison with such population data.

### DUOXA2 mutations

**FIG. 3.**
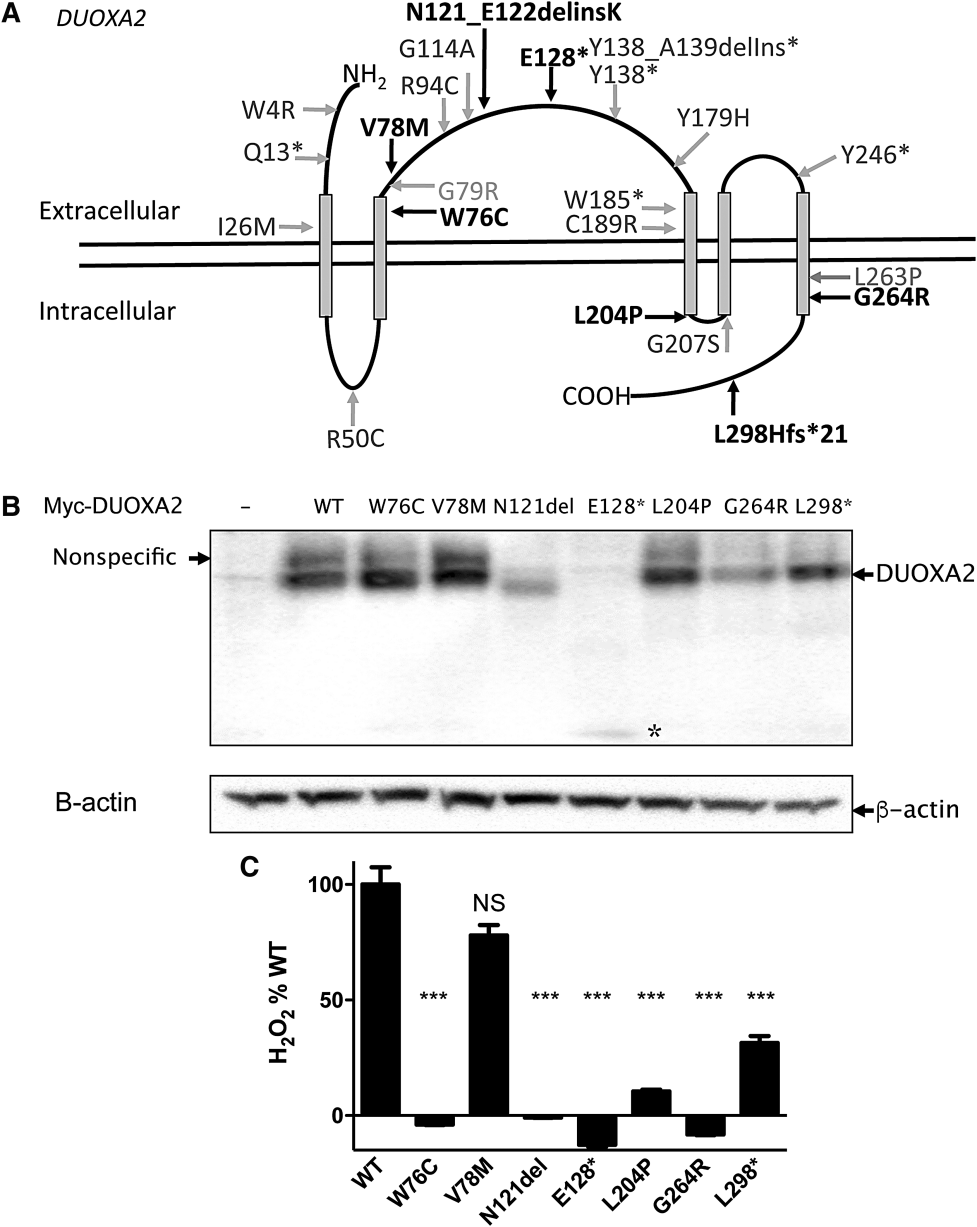
(**A**) DUOXA2, functional domains, and the position of mutations identified in this study (black) and mutations previously described in the literature (gray). The model was constructed using data from the UniProt database. (**B**) Western blot showing cell extracts from HEK293 cells transfected with myc-*DUOXA2* (wild-type or mutant) as indicated, with β-actin as loading control. A weak, nonspecific band at the same molecular weight as wild-type DUOXA2 is also detectable. −, cells transfected with empty vector; N121del, p.N121_E122delinsK; L298*, p.L298Hfs*21, * p.E128*. (**C**) Hydrogen peroxide (H_2_O_2_) production by the DUOX2–DUOXA2 complex in HeLa cells co-transfected with *DUOX2* and either wild-type (WT) or mutant *DUOXA2*. Data shown were taken one hour after the addition of Amplex Red reagent and are expressed as % WT. Compared with WT, NS, *p* > 0.05; ***p < 0.0005; one-way analysis of variance (ANOVA) with Tukey's *post hoc* test. Bars denote standard error of the mean. H_2_O_2_ production by p.W76C, p.N121_E122delinsK, p.E128*, p.L204P, and p.G264R was not significantly different from control cells (one-way ANOVA with Tukey's *post hoc* test).

Seven novel *DUOXA2* mutations were identified with MAF <0.001 in ExAC, in six cases of Asian (*n* = 4), white (*n* = 1), and American African (*n* = 1) ethnicity (Fig. 3A and Supplementary Tables S1 and S3). Four cases had monoallelic mutations (p.E128*, p.V78M, N121_E122delinsK) and two cases had compound heterozygous mutations ([p.L298Hfs*21;p.G264R], or [p.W76C; p.L204P]).

Only four of the reported *DUOXA2* missense and nonsense mutations have been characterized (9,13,18,19). Therefore, the functional consequences of all the mutations identified in this study were investigated. HEK293 cells were transfected with NH_2_-terminal c-Myc epitope tagged *DUOXA2* (14), and expression of the missense, nonsense, and frameshift DUOXA2 mutant proteins was assessed using Western blot analysis. All the mutant proteins were detected, although expression of p.E128* appeared decreased. DUOXA2 is a 34.8 kDa protein that is thought to undergo N-glycosylation at three consensus sites: N84, N109, and N121 (14). The deletion–insertion mutation, (p.N121_E122delinsK), abolishes the N121 glycosylation site, resulting in migration of the mutant protein as an incompletely glycosylated, lower molecular weight band (Fig. 3A,B).

DUOXA2 is required both for expression of DUOX2 at the plasma membrane, and to facilitate DUOX2-mediated H_2_O_2_ production (14,20). *DUOX2*-mediated H_2_O_2_ production in HeLa cells transfected with *DUOX2* alone was indistinguishable from background levels, while co-transfection with wild-type *DUOXA2*-myc/His resulted in a significant increase of H_2_O_2_ production. All mutant *DUOXA2* constructs, except for the V78M and L298Hfs*21 *DUOXA2* mutants, exhibited complete loss of function (Fig. 3C). L298Hfs*21 DUOXA2 was also deleterious but exhibited ∼30% wild-type activity. Although p.V78M DUOXA2 had a tendency to mild functional impairment, this was not significant, and the clinical implications of this variant are equivocal. Notably, venous thyroid function was less severely perturbed with this mutation than with the other variants (vTSH 29.3 mIU/L, fT4 11.8 pmol/L).

## Discussion

This study is the first to investigate the frequency of *DUOX2* or *DUOXA2* mutations in a borderline GIS CH cohort with a first bsTSH between 6 and 19.9 mIU/L, unlike previous studies that have studied cases recruited on the basis of higher bsTSH levels or known organification defect (10,12). Of the 52 infants evaluated, 50% harbored a *DUOX2/DUOXA2* variant that was either known or likely to be pathogenic. The observations suggest that *DUOX2*/*DUOXA2* mutations are a more common cause of borderline CH than anticipated and are consistent with the high frequency of defective iodide organification, likely due to underlying *DUOX2*/*DUOXA2* defects, in cases with borderline CH (TSH <15 mIU/L), documented in a previous Italian study (7). Since the patients exhibited only mild bsTSH elevation at screening yet have “true” dyshormonogenic CH with a recognized genetic basis, the findings may also inform debate on biochemical thresholds in national CH screening programs. Almost half the mutation-positive cases in this study would have escaped identification using a screening bsTSH cutoff of >10 mIU/L, although the majority had moderate to severe CH on venous testing, with a mean vTSH well above 25 mIU/L. Future studies are therefore needed to evaluate outcomes in treated versus untreated cases.

Similar proportions of the mutation-positive (54%) and mutation-negative (38%) cohorts had transient CH or were being maintained on a reducing dose of LT4 by the end of the study. The observation that maternal *DUOX2* mutation carriers were euthyroid in adulthood also supports transiency of CH in the majority of affected cases. Higher TSH screening cut points 20 years ago may have resulted in failure to diagnose borderline CH in these carrier parents. Transient CH is frequently reported in cases with DUOX2 and DUOXA2 deficiency and may reflect compensatory H_2_O_2_ production from DUOX1 and DUOXA1, resulting in euthyroidism when peak demand for thyroid hormone biosynthesis declines later in childhood (15). However, a comparable proportion (50%) of unselected cases of mild hyperthyrotropinemia with GIS may also exhibit transient hypothyroidism, and in this cohort, LT4 dose was also similar in mutation-positive and -negative cases, being around 50% of the usual doses for severe CH in each group (21). Therefore mutational screening of *DUOX2/DUOXA2* is not a sensitive predictor of the long-term severity of borderline CH and does not currently permit the identification of cases who will or will not benefit from LT4 treatment. The requirement for treating transient CH also remains ambiguous, although current guidelines recommend treatment of CH in infancy, with a trial off LT4 at three years of age in likely transient CH cases, when thyroxine-dependent central nervous system myelination is complete (22).

The benefits and adverse consequences of detecting borderline CH are frequently debated. This study did not seek to define the optimal bsTSH screening cutoff for CH, since the subgroup of borderline CH cases was investigated, with subsequent vTSH levels >25 mIU/L (∼10% of all cases with an initial borderline bsTSH result) (23). However, the study demonstrates that a high percentage of these cases, especially those harboring mutations in *DUOX2* or *DUOXA2*, exhibit markedly subnormal fT4 levels at an age when the developing brain is highly vulnerable to thyroid hormone deficiency, regardless of whether it is transient (24). This contrasts with mild neonatal hyperthyrotropinemia due to heterozygous *TSHR* mutations, in which TSH levels and thyroid status remain stable and may not require treatment (25). In this study, the early rise in bsTSH and more significantly elevated vTSH levels distinguished the *DUOX2/DUOXA2* mutation-positive cases from the mutation-negative cases. Further studies are needed to determine the duration of this early overt hypothyroidism, which has previously been reported in association with *DUOX2* or *DUOXA2* mutations and which eventually resolves (12). If prolonged, this may support the argument for diagnosing and treating *DUOX2*/*DUOXA2*-deficient cases. The mechanism for this evolving hypothyroidism following borderline bsTSH elevation is unclear. It is speculated that during the first two neonatal weeks, the ability of the thyroid to compensate for *DUOX2* deficiency may decline. To the best of the authors' knowledge, expression levels of *DUOX1*/*DUOXA1* and *DUOX2*/*DUOXA2* have not been characterized in the early neonatal period, but if the expression and functional importance of *DUOX2* were to increase relative to *DUOX1* shortly after birth, this may result in decompensated hypothyroidism in cases harboring *DUOX2*/*A2* mutations due to a failure of *DUOX1* to compensate for the defect.

Although thyroid hormone biosynthesis declines during later childhood, it increases markedly again during puberty and pregnancy, with maternal euthyroidism being critical for normal fetal neurodevelopment (26). The course of transient CH due to biallelic *DUOX2* mutations during puberty has only been evaluated in a Japanese population, and although individuals remained euthyroid, their high dietary iodine intake may confound comparison with British cases with differing iodine status (27). No studies have been performed to assess recurrence of transient CH during pregnancy. Anecdotal reports describe fluctuating thyroid status over time with DUOX2 dysfunction (28), suggesting that both patients and mutation-carrying relatives should be counseled regarding possible future risk of hypothyroidism, particularly during pregnancy when iodine status can also be compromised. Indeed, one maternal DUOXA2 carrier in our cohort developed non-autoimmune hypothyroidism postpartum.

Ethnic background, consanguinity, and sex did not differ between the *DUOX2/DUOXA2* mutation-positive, mutation-negative, and untested groups, but infants with CH from Asian backgrounds were overrepresented. Neonatal bsTSH levels reportedly exceed bsTSH thresholds more frequently across Indian subcontinent and Chinese populations, suggesting ethnic differences in thyroid physiology (29), and both *DUOX2* and *DUOXA2* mutations have most frequently been reported in East Asian populations (10,11). In this study, DUOX2 p.Q570L occurred frequently in South Asian individuals, and its background allele frequency of 0.01 in this ethnic group suggests that it may contribute more significantly to CH in the wider Asian population. Such high mutation frequency, but in the heterozygous state, argues against consanguinity being a major driver for the mutation occurrence. It is tempting to speculate that *DUOX2* and *DUOXA2* mutations confer beneficial effects in particular populations, protecting the thyroid gland from effects of excess iodine intake in the East Asian diet. Conversely, in the relatively iodine-deficient British population (30), low iodine status may synergize with the *DUOX* pathway mutations, culminating in dyshormonogenesis.

Despite recruitment on the basis of similar bsTSH values, the patients with *DUOX2* mutations exhibited biochemical heterogeneity on vTSH measurement. Complete lack of functional DUOX2 (e.g., due to homozygous p.Q202Tfs*99 mutations) resulted in fT4 levels ranging from 4.65 to 11.2 pmol/L, suggesting relative preservation of thyroid hormone biosynthesis in some cases, as has been observed previously with other homozygous DUOX2 truncation defects (17). This may reflect variable compensation by other thyroidal H_2_O_2_-producing enzymes (e.g., DUOX1), influence of other genetic variants affecting thyroid hormone biosynthesis, or differences in dietary iodide intake (31). Variable penetrance, with significant inter- and intrafamilial variability in associated phenotype, is characteristic of *DUOX2* mutations, and even biallelic truncating mutations may cause both mild transient and severe permanent CH (15). Indeed, although biallelic *DUOX2* mutations were initially thought to result in permanent CH, and monoallelic mutations to cause transient CH, the plethora of *DUOX2* mutations described to date refute this pattern in ∼40% cases (15). *DUOX2* mutations are typically associated with goitrous dyshormonogenesis, but a resistance to TSH phenotype has also been described (28). This poor genotype–phenotype correlation compounds prediction of CH severity and LT4 requirements on the basis of the *DUOX2* mutation or degree of functional impairment, and both environmental (e.g., dietary iodine) and genetic modifiers, including triallelic defects, have been reported to modulate the phenotype (10,12).

A surprisingly high percentage (11.5%) of cases harboring *DUOXA2* mutations in this borderline CH cohort suggests that mutations in this gene may be more significant in the etiology of CH than previously anticipated. Previous studies screening for *DUOXA2* defects have generally been undertaken in goitrous or biochemically unselected GIS CH (9,13). The data suggest that borderline GIS CH, with more elevated vTSH than bsTSH and subnormal venous fT4 levels, may be sensitive parameters for detecting *DUOXA2* mutation-positive CH. Although biochemically heterogeneous, both these and previously reported *DUOXA2* mutation cases result in transient CH or mild persistent hyperthyrotropinemia/CH. In this small cohort, cases with *DUOX2* and *DUOXA2* mutations were clinically and biochemically indistinguishable.

It is postulated that both the heterozygous and biallelic variants identified in this study caused CH. Although monoallelic *DUOX2* and *DUOXA2* variants may occur in the general population, borderline transient CH may evade diagnosis on neonatal CH screening, such that apparently healthy mutation carriers satisfy criteria for inclusion in population genetic databases such as ExAC. Where evaluated, previously reported heterozygous *DUOX2* mutations have been thought to confer a phenotype due to haploinsufficiency as a result of decreased plasma membrane expression, with preserved or absent intrinsic H_2_O_2_-generating activity, although dominant negative activity has been excluded for only a small minority of variants (32–34). A single evaluated heterozygous DUOXA2 loss-of-function mutation (p.I26M) did seem to result in dominant negative activity (13). This study did not evaluate in detail the mechanism by which the monoallelic mutations identified resulted in functional impairment, nor can a contribution from non-coding region (e.g., deep intron or regulatory region) variants or exon-level deletions in the opposite allele be excluded. It is not possible to comment on whether CH had an oligogenic basis, involving variants in *DUOX1* or *DUOXA1* or other genes involved in thyroid hormonogenesis.

DUOXA2–DUOX2 structure, function, and interaction remain poorly understood (14,35). Abolition of H_2_O_2_ synthesis with *DUOXA2* mutations N121_E122delinsK, W76C, L204P, and G264R attests to the functional importance of these residues, and the N121_E122delinsK mutant supports a functional requirement for normal glycosylation of this protein.

This study indicates that use of current, recommended cutoffs for bsTSH in screening for CH would fail to identify individuals with true dyshormonogenetic CH due to *DUOX2* and *DUOXA2* mutations, with *DUOX2*/*DUOXA2* cases comprising 50% of the borderline CH cohort. Furthermore, borderline TSH elevation in such cases is associated with overt thyroid hormone deficiency, which coincides with known maximal thyroxine dependency of brain development in the neonatal period. It is suggested that targeted *DUOX2* and *DUOXA2* sequencing in a borderline CH cohort will have a high diagnostic yield. These findings might argue for a lowering of bsTSH thresholds, but follow-up studies are required to assess whether cases with borderline bsTSH with *DUOX2*/*DUOXA2* mutations will benefit from an early diagnosis and subsequent LT4 treatment.

## Supplementary Material

Supplemental data

Supplemental data

Supplemental data

Supplemental data
