# Supplementary material for: DUOX2/DUOXA2 Mutations Frequently Cause Congenital Hypothyroidism that Evades Detection on Newborn Screening in the United Kingdom
Source: Thyroid. 2019 Jun 3;29(6):790–801. doi: 10.1089/thy.2018.0587 (PMC6588112; doi:10.1089/thy.2018.0587)
Supplement: Supplemental data [file Supp_Data.pdf]

Supplementary Data

Supplementary Methods

Between January 1, 2013, and December 31, 2015, 361,839 babies were screened for CH by the North Thames newborn screening laboratory based at Great Ormond Street Hospital (GOSH). The lower borderline bsTSH cut point was set at 6 mIU/L. A total of 519 infants with a gestational age ≥32 weeks were subsequently referred to the GOSH CH service. At review, venous thyroid function, antibody status, and maternal TFT were recorded for each child with demographic data, including ethnicity. Ethnicity was coded using the Ethnic Category Code published by the Health and Social Care Information Centre, United Kingdom. The position of the thyroid gland was established with Tc-99m scanning or thyroid ultrasound.

Assays

bsTSH concentrations were measured by the automated dissociation enhanced lanthanide fluoro immuno assay system (AutoDELFIA; PerkinElmer). The method is calibrated using six standards with values ranging from <1 mIU/L to 300 mIU/L, with some variation for each kit batch. The inter-batch precision of this method ranges from 6% to 8%, with no trend over the range of values. TSH in dried bloodspot specimens has been shown to be stable for at least one month at room temperature. If stored at +4°C with desiccant, there is no degradation of TSH for at least one year. All samples in this study were analyzed within one month of sample collection. There was no modification or change to the assay procedure during the study period, and no assay drift was reported. The assay was enrolled in the UK External Quality Assurance Scheme, and reports were satisfactory throughout this period.

Study criteria

Infants with an initial borderline TSH screening result between 6 and 19.9 mIU/L and a second bsTSH screen >6 mIU/L one week later were selected. Further inclusion criteria included initial venous TSH >25 mIU/L, negative thyroid antibody testing, a normally positioned thyroid gland, and treatment with T4.

At the time of the study period, infants of <32 weeks' gestation entered a different screening pathway and were therefore excluded from this study. Infants were also excluded with significant comorbidities ($n = 8$) or maternal and infant antibodies ($n = 1$). The characteristics of these excluded children are summarized below:

Reason for exclusion	N = *9*
Chromosome 13	1
Familial glucocorticoid deficiency and multiple congenital abnormalities	1
Kabuki syndrome	1
Trisomy 21	3
Vacterl	1
Vein of Galen	1
Positive maternal and infant antibodies	1

Of the 519 children referred to the service in this study time period, 83 children were eligible for study inclusion based on the criteria outlined above, and 52 infants were recruited. Eleven infants were followed up elsewhere, one family declined, and 19 were not able to provide the parental consent and/or DNA samples in the time frame of the study.

DUOX2 was screened in all cases, and in cases where no mutation was identified, the study proceeded to *DUOXA2* sequencing.

Genetic analyses

Mutations in *DUOX2* were classified as pathogenic if there was supporting literature, or if they affected the protein coding sequence (transcript ablation, stop gained, stop lost, stop retained, splice donor variant, splice acceptor variant, frameshift, inframe insertion, inframe deletion, initiator codon variant, splice region variant and missense variants) and were classified as at least ''possibly pathogenic'' by either SIFT or PolyPhen-2, using the transcript-specific predictions supplied in Ensembl, if predictions available), or ''disease-causing'' by MutationTaster (S1–S3), and had MAF in the ExAC database ≤0.02. Novel mutations were those not present in HGMD Professional or detected during a PubMed search for genetic studies in CH. *DUOXA2* mutations were assessed *in vitro* for their effect on *DUOX2*-mediated H_2O_2 production.

Mutagenesis

Receptor mutations were generated by site-directed mutagenesis of WT human *DUOXA2* cDNA and cloned into a eukaryotic expression vector (pcDNA3.1) downstream of a Myc tag or upstream of a Myc/His tag. HA-epitope tagged *DUOX2* and carboxy-terminal Myc/His-epitope tagged *DUOXA2* were a kind gift from Dr. S. Refetoff (S4).

H_2O_2 generation by the DUOX2–DUOXA2 *complex in HeLa cells*

HeLa cells, cultured on a 96-well plate in DMEM +10% FBS, PSF, and L-glutamine, were transfected using Lipofectamine 2000 (Thermo Fisher Scientific) with 50 ng HA-*DUOX2* and 25 ng *DUOXA2*-Myc/His expressing plasmids in combination with 50 ng BOS β-Gal plasmid. Extracellular H_2O_2 accumulation was assayed by the addition of Amplex Red reagent (Thermo Fisher Scientific) to the medium (50 μL/well: 1 × PBS with 1 nM Hepes, 0.25 μL Amplex Red stock, 0.5 μL HRP, 0.5 μL ionomycin 1 M) and measurement of fluorescence using an Infinite M1000 Pro microplate reader (Tecan). Values were adjusted for transfection efficiency quantified by a β-Gal assay. Results are an average of at least 10 experiments, presented with wild-type *DUOX2–DUOXA2* set as 100%. Data were analyzed, and figures were generated using

GraphPad Prism version 5.0d for Mac OS X November 8th 2010 (GraphPad Software).

DUOXA2 expression analysis by Western blot

HEK293 cells, cultured on a six-well plate in DMEM +10% FBS, PSF, and L-glutamine, were transfected with Myc-*DUOXA2*, 1 μg plasmid, using Lipofectamine 2000 (Thermo Fisher Scientific). Cell were lysed (200 nM NaCl, 20 mM Tris HCl pH 7 [NaOH], 1 mM DTT, 1% NP40, 2% SDS, complete protease inhibitor cocktail, 10 IU/mL benzonase [Novagen]), and 50 μg of each were run on a 10% SDS-PAGE gel (Thermo Fisher Scientific). Expression of *Duoxa2* was analyzed by Western blotting using an anti-Myc antibody (9E10; Santa Cruz Biotechnology).

Supplementary References

S1. Schwarz JM, Cooper DN, Schuelke M, Seelow D 2014 MutationTaster2: mutation prediction for the deep-sequencing age. Nat Methods **11:**361–362.

S2. Adzhubei IA, Schmidt S, Peshkin L, Ramensky VE, Gerasimova A, Bork P, Kondrashov AS, Sunyaev SR 2010 A method and server for predicting damaging missense mutations Nat Methods **7:**248–249.

S3. Kumar P, Henikoff S, Ng PC 2009 Predicting the effects of coding non-synonymous variants on protein function using the SIFT algorithm. Nat Protoc **4:**1073–1081.

S4. Grasberger H, Refetoff S 2006 Identification of the maturation factor for dual oxidase. Evolution of an eukaryotic operon equivalent. J Biol Chem **281:**18269–18272.
