# Supplementary material for: DUOX2/DUOXA2 Mutations Frequently Cause Congenital Hypothyroidism that Evades Detection on Newborn Screening in the United Kingdom
Source: Thyroid. 2019 Jun 3;29(6):790–801. doi: 10.1089/thy.2018.0587 (PMC6588112; doi:10.1089/thy.2018.0587)
Supplement: Supplemental data [file Supp_Table1.pdf]

SUPPLEMENTARY TABLE S1. CLINICAL, DEMOGRAPHIC, AND BIOCHEMICAL CHARACTERISTICS OF THE CASES HARBORING *DUOX2* AND *DUOXA2* MUTATIONS

Case (sex)	*UK ONS*	*Consanguinity*	*Guthrie TSH 1 (ref. range <6 mIU/L)*	*Guthrie TSH 2 (ref. range <6 mIU/L)*	*Venous TSH (ref. range <6 mIU/L)*	*Venous fT4 (ref. range 12.5–24.6 pmol/L)*	*Technetium scan*	*Outcome of CH/dose of levothyroxine*
Cases harboring DUOX2 mutations								
1 (M)	R Chinese	N	6.6 (5)	14.8 (14)	58.4 (20)	7.3	NA	Transient
2 (M)	R Chinese	N	11.8 (5)	16 (9)	54.0 (15)	9.4	Avid uptake	Transient
3 (F)	K Asian	Y	18.2 (6)	17.6 (13)	47.6 (18)	15.8	Normal location	Reducing dose of LT4
4 (F)	K Asian	N	7.5 (5)	8.9 (13)	30.1	15.7	Avid uptake	2.13 μg/kg at 4 years
5 (F)	A White	N	13.6 (5)	10.8 (12)	36.4 (19)	10.9	Avid uptake	Transient
6 (F)	N Black	N	17.4 (6)	70.7 (22)	76.9 (28)	4.8	Avid uptake	Transient
7 (M)	A White	N	9.9 (5)	12 (10)	34.0 (16)	12.0	Reasonable uptake	Transient
8 (F)	L Asian	N	18.8 (5)	40 (11)	150 (18)	8.1	Avid uptake	1.85 μg/kg at 3.5 years
9 (F)	J Asian	Y	8.0 (5)	34.5 (15)	69.0 (18)	6.2	Avid uptake	Transient
10 (M)	S Asian	N	7 (11)	10.5 (16)	36.5 (21)	12.5	Avid uptake	Reducing dose of LT4
11 (F)	K Asian	N	12.7 (6)	37.1 (13)	146 (20)	<3.9	Avid uptake	Transient
12 (F)	L Asian	N	9.3 (8)	16.2 (19)	29.8 (24)	12.8	NA	2.25 μg/kg at 2.3 years
13 (M)	H Asian	N	6.2 (5)	12.6 (13)	45.2 (19)	14.8	Normal uptake	1.6 μg/kg at 2.75 years
14 (M)	R Other	N	8.8 (6)	13.3 (12)	80.1 (20)	3.9	Avid uptake	1.1 μg/kg at 2.5 years
15 (M)	R Chinese	N	9.6 (6)	28.5 (15)	35.8	8.8	Avid uptake	Transient
16 (M)	K Asian	Y	12.3 (5)	25.2 (13)	92.8	4.65	Normal uptake	0.8 μg/kg at 1.75 years
17 (M)	R Chinese	N	8.2 (5)	17.5 (12)	85.7	6	Avid uptake	2.7 μg/kg at 1.3 years
18 (F)	H Asian	N	11.7 (5)	46.6 (10)	95.81	11.3	NA	1.71 μg/kg at 1.8 years
19 (F)	J Asian	N	8.4 (5)	7.9 (13)	51.5	12.5	Avid uptake	2.95 μg/kg at 1.3 years
20 (M)	F Mixed	N	12.9 (5)	—	65.9	9.6	NA	4.20 μg/kg at 1.5 years
Cases harboring DUOXA2 mutations								
21 (M)	J Asian	N	10.9 (4)	24.1 (11)	62.1 (16)	9.4	Avid uptake	Reducing dose of LT4
22 (M)	K Asian	N	13.1 (6)	25 (15)	101.0 (23)	6.5	Avid uptake	Transient
23 (F)	S Mixed	N	12.3 (5)	25.6 (13)	101.0 (18)	8.7	Avid uptake	2.7 μg/kg at 4.2 years
24 (M)	K Asian	N	14.1 (6)	12.5 (13)	29.3 (16)	11.8	Avid uptake	Transient
25 (F)	A white	N	7.9 (5)	14.3 (12)	48.2 (18)	9.2	Avid uptake	Reducing dose of LT4
26 (M)	H Asian	N	14.6 (4)	34.6 (12)	125.0 (18)	5.5	Avid uptake	NA

Ethnicity is recorded according to the UK Office for National Statistics (ONS) coding at newborn screening. Where available, age in days at sampling is denoted in parentheses after hormone measurements. "Avid uptake" is uptake above the normal range, which is defined as 0.45–1.7%.
