# Supplementary material for: DUOX2/DUOXA2 Mutations Frequently Cause Congenital Hypothyroidism that Evades Detection on Newborn Screening in the United Kingdom
Source: Thyroid. 2019 Jun 3;29(6):790–801. doi: 10.1089/thy.2018.0587 (PMC6588112; doi:10.1089/thy.2018.0587)
Supplement: Supplemental data [file Supp_Table3.pdf]

Supplementary Table S3. Summary of *DUOX2* and *DUOXA2* Mutations Identified in this Study

Case	*cDNA*	*Protein*	*Inheritance*	*Zygosity*	*Known/Novel*	*AF (ExAC)*	*Max. AF ExAC (Ethnicity)*	*SIFT*	*PolyPhen*
DUOX2 *mutations*									
1 (M)[a]	*c.1232G>A*	*p.R411K*	M	Heterozygous	*Known*	0.00005	0.0007 (East Asian)	Del	Poss D
	c.3251G>A	*p.R1084Q*	P	Heterozygous	*Known*	0.00007	0.0002 (East Asian)	Del	Prob D
2 (M)	*c.3693+1G>T*	NA	M	Heterozygous	*Known*	0.0001	0.002 (East Asian)	NA	NA
3 (F)	c.2895_2898delGTTC	p.F966Sfs*29	M	Heterozygous	Known	0.003	0.01 (European Finnish)	NA	NA
4 (F)	c.1709A>T	p.Q570L	NA	Heterozygous	Known	0.003	0.01 (South Asian)	Del	Prob D
	c.2895_2898delGTTC	p.F966Sfs*29	NA	Heterozygous	Known	0.003	0.01 (European Finnish)	NA	NA
5 (F)	**c.385C>T**	**p.L129F**	M	Heterozygous	**Novel**	0.00005	**0.00009 (European non-Finnish)**	T	B
6 (F)	*c.2290C>T*	*p.R764W*	M	Heterozygous	*Known*	0.0004	0.004 (African)	Del	Prob D
7 (M)	c.2895_2898delGTTC	p.F966Sfs*29	M	Heterozygous	Known	0.003	0.01 (European Finnish)	NA	NA
8 (F)	c.1709A>T	p.Q570L	M, P	Homozygous	Known	0.003	0.01 (South Asian)	Del	Prob D
9 (F)	c.602dupG	p.Q202Tfs*99	M, P	Homozygous	Known	0.001	0.0031 (Latino)	NA	NA
10 (M)	c.2635G>A	p.E879K	M	Heterozygous	Known	0.00005	0.0006 (East Asian)	Del	Prob D
11 (F)	c.1709A>T	p.Q570L	M	Heterozygous	Known	0.003	0.01 (South Asian)	Del	Prob D
12 (F)[a]	**c.715G>A**	**p.A239T**	M	Heterozygous	**Novel**	—	—	T	B
	c.1709A>T	p.Q570L	P	Heterozygous	Known	0.003	0.01 (South Asian)	Del	Prob D
13(M)[a]	c.1709A>T	p.Q570L	P	Heterozygous	Known	0.003	0.01 (South Asian)	Del	Prob D
	c.4405G>A	*p.E1469K*	M	Heterozygous	*Known*	0.00006	0.0003 (South Asian)	Del	Prob D
14 (M)	c.1462G>A	p.G488R	M	Heterozygous	Known	0.0002	0.002 (South Asian)	Del	Prob D
15 (M)	*c.2048G>T*	*p.R683L*	P	Heterozygous	*Known*	0.0003	0.004 (East Asian)	Del	Prob D
	c.4027C>T	*p.L1343F*	P	Heterozygous	*Known*	0.0005	0.007 (East Asian)	Del	Poss D
16 (M)	c.2895_2898delGTTC	p.F966Sfs*29	NA	Homozygous	Known	0.003	0.01 (European Finnish)	NA	NA
17 (M)	c.605_621del17	p.Q202Rfs*93	P	Heterozygous	Known	—	—	NA	NA
18 (F)	c.602dupG	p.Q202Tfs*99	M, P	Homozygous	Known	0.001	0.0031 (Latino)	NA	NA
19 (F)[a]	c.1709A>T	p.Q570L	M	Heterozygous	Known	0.003	0.01 (South Asian)	Del	Prob D
	**c.3940G>A**	**p.E1314K**	P	Heterozygous	**Novel**	0.00002	0.00006 (South Asian)	Del	Prob D
20 (M)	*c.2894C>T*	*p.S965L*	NA	Heterozygous	*Known*	0.0001	0.0005 (East Asian)	Del	B
DUOXA2 *mutations*									
21 (M)	**c.382G>T**	**p.E128***	P	Heterozygous	**Novel**	0.00002	0.0001 (South Asian)	NA	NA
22 (M)	**c.363_365delCGA**	**p.N121_E122delinsK**	NA	Heterozygous	**Novel**	—	—	Del[a]	Prob D[a]
23 (F)[a]	**c.790G>C**	**p.G264R**	P	Heterozygous	**Novel**	—	—	NA	NA
	**c.893_894delTT**	**p.L298Hfs*21**	M	Heterozygous	**Novel**	0.0009	0.0099 (African)	NA	NA
24 (M)	**c.232G>A**	**p.V78M**	NA	Heterozygous	**Novel**	0.00005	0.0004 (South Asian)	Del	Prob D
25 (F)[a]	**c.228G>T**	**p.W76C**	P	Heterozygous	**Novel**	0.00005	0.00008 (Latino)	Del	Prob D
	**c.611T>C**	**p.L204P**	M	Heterozygous	**Novel**	0.00005	0.00009 (European, non-Finnish)	Del	Poss D
26 (M)	**c.382G>T**	**p.E128***	M	Heterozygous	**Novel**	0.00002	0.0001 (South Asian)	NA	NA

Novel mutations shown in bold; previously described in association with CH but not functionally characterized shown in italics; previously described in association with CH and functionally characterized or truncating shown in normal type.

[a]Cases who were compound heterozygous for two different mutations. All mutations were predicted to be disease-causing by MutationTaster.

SIFT: Del, deleterious; T, tolerated; NA, not available (non-single amino acid change); AF Exac, total allele frequency (Exome Aggregation Consortium [ExAC], Cambridge, MA; http://exac.broadinstitute.org); Max.AF ExAC, maximal allele frequency (ExAC), with the corresponding ethnicity in parentheses. PolyPhen: Prob D, probably damaging; Poss D, possibly damaging; B, benign. Inheritance: P, paternal; M, maternal; NA, not available.
