# Supplementary material for: DUOX2/DUOXA2 Mutations Frequently Cause Congenital Hypothyroidism that Evades Detection on Newborn Screening in the United Kingdom
Source: Thyroid. 2019 Jun 3;29(6):790–801. doi: 10.1089/thy.2018.0587 (PMC6588112; doi:10.1089/thy.2018.0587)
Supplement: Supplemental data [file Supp_Table2.pdf]

Supplementary Table S2. Clinical, Demographic, and Biochemical Characteristics of Mutation Negative Cases

Case	*UK ONS*	*Consanguinity*	*Guthrie TSH 1 (ref. range <6 mIU/L)*	*Guthrie TSH 2 (ref. range <6 mIU/L)*	*Venous TSH (ref. range <6 ImU/L)*	*Venous fT4 (ref. range 12.5–24.6 pmol/L)*	*Technetium scan*	*Dose LT4*
27 (M)	N Black	N	15.6 (13)	11 (18)	35.1 (21)	12.4	Avid uptake	Transient
28 (F)	H Asian	N	6.8 (4)	23.9 (10)	76.8 (18)	<3.9	Avid uptake	Transient
29 (M)	A White	N	13.8 (6)	27.3(13)	100.0 (20)	4.3	Avid uptake	Transient
30 (F)	H Asian	N	17.8 (5)	10.6 (11)	38.6 (18)	13.6	Low uptake	4.73 μg/kg at 3.8 years
31 (M)	A White	N	6.8 (5)	11.2 (8)	33.9 (16)	10.2	Avid uptake	1.58 μg/kg at 3.8 years
32 (F)	G Mixed	N	10.0 (5)	9.6 (18)	35.3 (26)	7.4	Avid uptake	Transient
33 (F)	H Asian	N	9.7 (5)	9.3 (2)	29.6 (12)	14.9	Poor uptake	1.3 μg/kg at 3.25 years
34 (M)	K Asian	N	14.6 (5)	34.9 (12)	75.3 (19)	10.5	Normal uptake	Transient
35 (M)	White	N	12.1 (5)	10.3 (20)	38.4 (26)	12.0	NA	2.1 μg/kg at 4 years
36 (F)	White	N	8.8 (5)	24.1 (12)	40.9 (19)	8.5	Avid uptake	Transient
37 (M)	J Asian	Y	12.3 (6)	21.9 (16)	55.6 (20)	8.5	Avid uptake	2.2 μg/kg at 3.25 years
38 (M)	L Asian	Y	11.5 (5)	21.6 (14)	34.6 (19)	10.3	Avid uptake	Transient
39 (F)	S Asian	N	18.3 (6)	16.2 (14)	27.5 (19)	13.9	Clearly visualized	Transient
40 (M)	J Asian	Y	15.1 (7)	17(14)	68.0 (20)	9.6	Avid uptake	1.45 μg/kg at 3 years
41 (F)	N Black	N	12.2 (5)	7.5 (15)	25	15.6	Poor uptake	1.5 μg/kg at 3.3 years
42 (M)	S Other	Y	10 (5)	10.6 (12)	32.3 (16)	12.2	Avid uptake	Transient
43 (M)	G Mixed	N	14.6 (5)	7.5 (12)	26.8 (18)	14.0	Avid uptake	1.86 μg/kg at 2.75 years
44 (F)	A white	N	10.4 (5)	8.3 (12)	41.1 (19)	12.0	Low uptake	2 μg/kg at 2.5 years
45 (M)	B White	N	8.5 (6)	11.3 (12)	35.2 (17)	8.1	Avid uptake	1.85 μg/kg at 2.5 years
46 (M)	NS Asian	N	12.6 (5)	17.4 (8)	47.4 (12)	12.4	Normal uptake	2.1 μg/kg at 2.4 years
47 (M)	L Asian	N	11.8 (7)	20.3 (12)	26.4	11.3	Avid uptake	1.55 μg/kg at 2.3 years
48 (M)	R Chinese	N	14.6 (5)	35 (13)	25.4	11.8	Low grade uptake	Transient
49 (M)	K Asian	N	15.7 (5)	30 (10)	60.6 (17)	11.5	Avid uptake	2.7 μg/kg at 2.1 years
50 (F)	Asian	Y	9.8 (6)	10 (13)	26.2	15.3	Reduced uptake	3.6 μg/kg at 1.75 years
51 (F)	K Asian	N	6.4 (5)	10.3	25	13	Avid uptake	2.14 μg/kg at 1.8 years
52 (F)	N Black	N	6.3 (5)	13.6 (12)	43.1	10.9	Avid uptake	1.8 μg/kg at 1.6 years

Ethnicity is recorded according to the UK ONS coding at newborn screening. Where available, age in days at sampling is denoted in parentheses after hormone measurements. ''Avid uptake'' is uptake above the normal range, which is defined as 0.45–1.7%.
